# An *In Vitro/In Vivo* Model to Analyze the Effects of Flubendazole Exposure on Adult Female *Brugia malayi*

**DOI:** 10.1371/journal.pntd.0004698

**Published:** 2016-05-04

**Authors:** Maeghan O’Neill, Abdelmoneim Mansour, Utami DiCosty, James Geary, Michael Dzimianski, Scott D. McCall, John W. McCall, Charles D. Mackenzie, Timothy G. Geary

**Affiliations:** 1 Institute of Parasitology, McGill University, Ste-Anne-de-Bellevue, Quebec, Canada; 2 TRS Labs, Athens, Georgia, United States of America; 3 Department of Pathobiology and Diagnostic Investigation, Michigan State University, East Lansing, Michigan, United States of America; Institute of Medical Microbiology, Immunology and Parasitology, GERMANY

## Abstract

Current control strategies for onchocerciasis and lymphatic filariasis (LF) rely on prolonged yearly or twice-yearly mass administration of microfilaricidal drugs. Prospects for near-term elimination or eradication of these diseases would be improved by availability of a macrofilaricide that is highly effective in a short regimen. Flubendazole (FLBZ), a benzimidazole anthelmintic registered for control of human gastrointestinal nematode infections, is a potential candidate for this role. FLBZ has profound and potent macrofilaricidal effects in many experimental animal models of filariases and in one human trial for onchocerciasis after parental administration. Unfortunately, the marketed formulation of FLBZ provides very limited oral bioavailability and parenteral administration is required for macrofilaricidal efficacy. A new formulation that provided sufficient oral bioavailability could advance FLBZ as an effective treatment for onchocerciasis and LF. Short-term *in vitro* culture experiments in adult filariae have shown that FLBZ damages tissues required for reproduction and survival at pharmacologically relevant concentrations. The current study characterized the long-term effects of FLBZ on adult *Brugia malayi* by maintaining parasites in jirds for up to eight weeks following brief drug exposure (6–24 hr) to pharmacologically relevant concentrations (100 nM—10 μM) in culture. Morphological damage following exposure to FLBZ was observed prominently in developing embryos and was accompanied by a decrease in microfilarial output at 4 weeks post-exposure. Although FLBZ exposure clearly damaged the parasites, exposed worms recovered and were viable 8 weeks after treatment.

## Author Summary

Onchocerciasis and lymphatic filariasis are debilitating diseases caused by infections with filarial nematodes. The World Health Organization aims to eliminate these infections as public health problems. Despite prolonged control efforts, including chemotherapy through mass drug administration (MDA), transmission and infections persist. Addition of a microfilaricide that is efficacious in a short regimen would enhance prospects for achieving elimination goals. We investigated the long-term effects of the macrofilaricidal drug, flubendazole (FLBZ), on *Brugia malayi*. Adult parasites were exposed in culture to FLBZ at pharmacologically relevant concentrations (100 nM—10 μM) for up to 24 hr prior to implantation into the abdominal cavity of a jird for long-term maintenance. The greatest drug effect was on embryogenesis; morphological damage was most evident in early developmental stages. There was also a decrease in the release of microfilaria (mf) from the adult. Interestingly, no damage was observed to fully formed mf. Although further studies are required to determine to what extent these findings can be extrapolated to a field setting, an exposure profile which may produce similar effects *in vivo* has been defined.

## Introduction

Infections with filarial parasites that cause lymphatics filariasis (LF) and onchocerciasis can lead to debilitating symptoms and cause great economic losses in endemic countries [1,2]. Control measures have relied on mass drug administration (MDA) of either ivermectin or diethylcarbamazine with albendazole since the Global Programme to Eliminate Lymphatic Filariasis (GPELF), the Onchocerciasis Control Programme (OCP) and the African Programme for Onchocerciasis Control (APOC) were created with the aim of eliminating LF and onchocerciasis as public health problems [3,4]. These drugs appear to act mainly as microfilaricides in an MDA setting that provides yearly dosing for an extended period of time to achieve elimination or local eradication [5]. With the recent decline in individuals reported to be infected with LF and onchocerciasis [6–9], the goal of elimination/control set by the World Health Organization [10] is closer to being achieved. Yet there remain a large number of individuals infected with these parasites. Additionally, MDA programmes for onchocerciasis within Africa are geographically limited due to severe adverse events associated with acute killing of *Loa loa* microfilaria (mf) in individuals bearing high parasitemia following treatment with ivermectin [11]. The introduction of a safe macrofilaricidal drug into control programs would is predicted to greatly enhance the ability to eliminate these infections in a timely manner.

Flubendazole (FLBZ), a benzimidazole (BZ) anthelmintic, is a candidate macrofilaricide for use in onchocerciasis and LF control programs. Initially introduced for treatment of infections of livestock animals with gastrointestinal (GI) parasitic nematode infections [12], FLBZ was subsequently approved for the same indication in humans [13], for which it is highly efficacious [14,15]. FLBZ has exhibited very high macrofilaricidal efficacy when administered parenterally in experimental filarial models [16–18] and in a human trial in onchocerciasis [19]. Unfortunately, available formulations of the drug afford very limited oral bioavailability. Additionally, the formulation used for parenteral dosing in the human onchocerciasis study (19) led to severe injection site reactions, and its development was not pursued.

Recent efforts have been made to re-formulate FLBZ to enable oral dosing [18,20,21]. Definition of the pharmacokinetic profiles needed for efficacy with an orally-bioavailable formulation would be facilitated by knowledge of the time-concentration exposure profiles at which FLBZ is detrimental to the survival of adult filariae. Previous data show that exposure to pharmacologically relevant concentrations of FLBZ or its bioactive reduced metabolite (R-FLBZ) in culture elicits damage to the hypodermis, developing embryos, and intestine of adult female *B*. *malayi*, but this damage is not accompanied by apparent changes in motility or viability [22]. Developing an exposure-efficacy profile in vitro can assist in the definition of target pharmacokinetic profiles for dose selection in advanced development. We adapted a transplant model for *B*. *malayi* in an effort to define a concentration of FLBZ that would be lethal after short-term exposures (≤ 1 day). The present study examined long-term concentration-dependent effects of exposure to FLBZ *in vitro* in *B*. *malayi* survival and viability after recovery from naïve jirds following transplantation.

## Methods

### Ethics statement

The transplant surgery was carried out under AUP 15–07 (2) and was approved by the TRS Labs Inc., Institutional Animal Care and Use Committee (IACUC).

### Animal care

Male jirds (*Meriones unguiculatus*) approximately 24–30 weeks of age (55–75 grams) were used as the source for and recipients of parasites in this study. The jirds were multiple housed (3-5/cage) in solid bottom, clear/translucent cages with bedding and wire mesh lids. The study room was maintained on a 12 hour light/dark cycle within a temperature range of 18–26°C and a relative humidity range of 30 to 70%. Jirds were fed ad libitum with an appropriate certified rodent diet and water was provided ad libitum by an automatic watering system and/or water bottles.

### Parasites

Adult male and female *B*. *malayi* were isolated from the peritoneal cavity of jirds >120 days post-infection as described [23,24]. Briefly, recovered adult worms were washed three times with warm (37°C) RPMI-1640 medium supplemented with 100 U/mL penicillin, 100 μg/mL streptomycin, and 0.4% gentamycin (Sigma-Aldrich Corp., St. Louis, MO, USA; hereafter referred to as RPMI).

Adult females were exposed to varying concentrations of FLBZ (10 μM, 1 μM, or 100 nM; Epichem Pty Ltd, Murdoch, WA, Australia) *in vitro* for 6, 12, or 24 hr. FLBZ solutions were prepared by dissolving the respective drug in 100% DMSO, with addition to RMPI to a final DMSO concentration of 0.1%. Control RPMI contained an equivalent concentration of DMSO. Following exposure, male and female worms (10–15 each) were rinsed with RPMI and then transplanted into the peritoneal cavity of naïve jirds as described [25]. The recipient jird was anesthetized with a 1:1:5 cocktail of xylazine:saline:ketamine and the fur was removed from the right ventral abdomen with electric clippers. The skin was wiped with 70% ethanol prior to making a 1 cm incision in the skin and body wall to expose the peritoneal cavity. Worms were aspirated into a Pasteur pipette which was inserted into the incision and the worms expressed into the peritoneal cavity. The incision was closed with Autoclip staples until necropsy 5 days, 4 weeks or 8 weeks later.

### Parasite viability

#### Recovery

At each end point, jirds were sacrificed and the peritoneal cavity opened for examination for adult worms. Worms were removed from the peritoneal cavity using forceps placed in RPMI. The carcass was then soaked in warm saline to allow remaining worms to enter the media and the additional worms were placed in the RPMI with the original batch of worms. The number and sex of adult *B*. *malayi* were recorded for each animal.

#### Motility

Parasite motility was assessed visually under light microscopy. Worms were scored as immotile, with no motion during the observation period; slightly motile, where only twitching of the head and/or tail was observed; moderately motile, with slow sinusoidal movements; or highly motile, comparable to parasites obtained from drug-free control jirds. Each sample was observed for at least one minute before scoring. Worms were fixed immediately thereafter as described [22] for subsequent histological analysis.

### Effects on embryogenesis

#### Microfilarial release

Microfilariae were obtained from the peritoneal cavity of jirds at the time of necropsy. A glass transfer pipet was used to transfer approximately10 ml of warm RPMI into the jird peritoneal cavity through an abdominal incision. After massaging the abdomen, the RPMI was withdrawn and transferred to a conical tube. The number of mf was counted in aliquots of 10 μl in triplicate.

#### Embryograms

Two to four worms from each jird in each treatment group were placed in a 1.5ml Eppendorf tube with 500 μl PBS, gently crushed with a pestle and vortexed for 5–10 sec. A 10 μl sample was transferred to each chamber of a haemocytometer to count the various embryonic stages following the method of Schultz-Key [26]. Embryos were classified into 6 stages: oocyte, early morula, late morula, S-shaped ‘sausage’ stage, coiled ‘pretzel’ stage, and stretched mf. A minimum of 100 embryos were counted to assess the proportional representation of each developmental stage.

### Histological preparation

*B*. *malayi* were fixed in glutaraldehyde (5% glutaraldehyde in 0.1 M sodium cacodylate buffer, pH 7.2) for a minimum of 48 hr in preparation for histological processing. Worms from each treatment were combined into groups and coiled prior to embedding in Histogel (FisherScientific; Pittsburgh, Pennsylvania, USA), which allowed visualization of various anatomical regions in multiple worms on a single slide. Dehydration, clearing, and vacuum infiltration with paraffin were completed using a Sakura VIP tissue processor. Parasites were then embedded in paraffin with the ThermoFisher HistoCentre III embedding station. A Reichert Jung 2030 rotary microtome was used to cut 4–5 micron sections, which were dried at 56°C for 2–24 hr. Slides were stained with haematoxylin and eosin prior to examination under light microscopy.

### Assessment of worm damage

Sections were assessed independently by three parasitologists familiar with filarial nematode morphology, including a board-certified pathologist/parasitologist (CDM), as described in [22]. Briefly, worms from two independent experiments were examined for damage to the body wall, including cuticle, hypodermis and longitudinal muscle; intestine; and reproductive tract, including the uterine wall and embryonic stages (classified as early [oocytes, early morulae, late morulae] or late [sausage, pretzel, microfilariae]); and the pseudo-coelomic cavity. For comparative analysis of drug-induced effects, tissues were classified into four categories: no damage (0), minor (1), moderate (2), or severe (3). The damage score was determined by assessing tissues for nuclear and cytoplasmic distortions, cellular size and shape, membrane integrity, accumulation of debris, and distortion of overall anatomical integrity.

Two methods of analysis were performed as previously described [22]. The first adhered to classical techniques used by histopathologists to determine tissue damage, in which all sections on a slide were surveyed, interpreted and translated into a single damage score for each tissue type. The second method involved scoring damage in each tissue type for each worm section on a slide. These scores were averaged for all sections on the slide to obtain the damage score.

### Statistical analysis

Statistical analyses were performed using the GraphPad Prism 6 package. Percent recovery, microfilarial abundance, embryogram, and histology results were analysed using a two-way ANOVA between treatment groups and time points. All statistical tests were interpreted at the 5% level of significance.

## Results

### Effect of FLBZ on adult worm viability

#### Recovery

**Fig 1:**
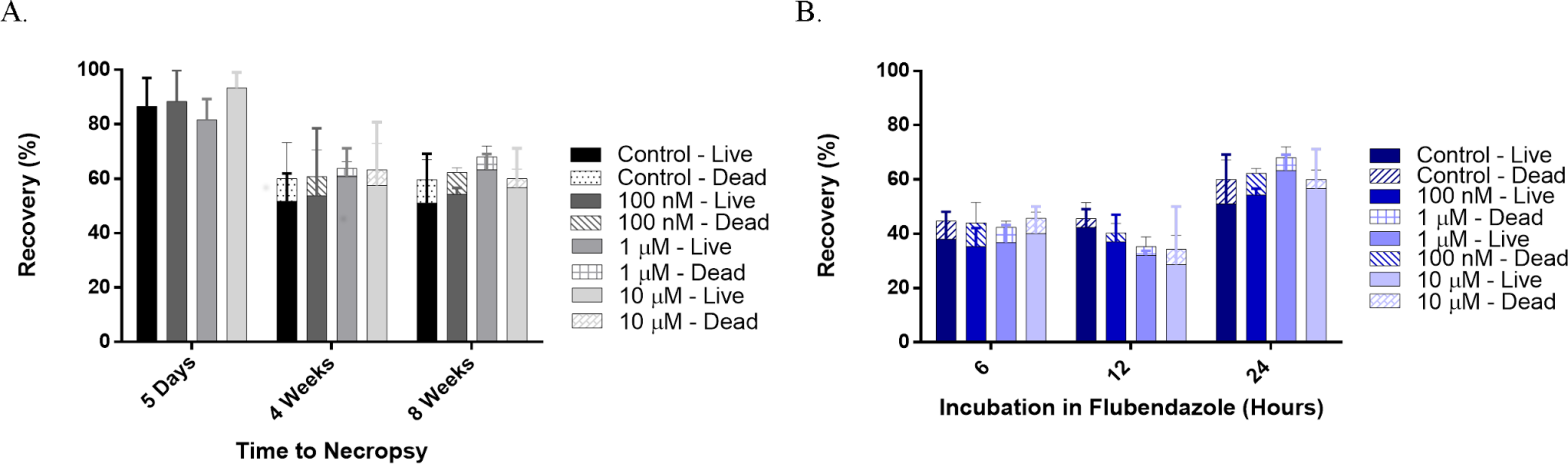
Effect of FLBZ on recovery of exposed and transplanted adult *B*. *malayi* male and female worms from the peritoneal cavity of naïve jirds. A. Five day, four week, or eight week maintenance in jirds after transplantation following 24 hr in culture. Data is a combination of two experiments. In experiment one 20 worms (10 male and ten female) were transplanted into each of three jirds for all treatment groups and maintained for 5 days or 4 weeks. Thirty worms (15 male and 15 female) were transplanted into each of three jirds per treatment group in experiment two and maintained for 4 weeks or 8 weeks. The 4 week data from each experiment did not differ significantly, therefore, they were combined for presentation purposes. B. Worm recovery eight weeks after transplantation following 6, 12, or 24 hr exposure to FLBZ *in vitro*. Thirty worms (15 male and 15 female) were transplanted into each of three jirds per treatment group, following the appropriate duration of *in vitro* exposure to FLBZ. Bars indicate the mean recovery of worms, both male and female, from each jird in a treatment group. Lines represent the standard deviation.

Flubendazole did not affect the recovery of adult worms following 24 hr exposure prior to transplant (Fig 1A). Percent recovery was > 80% in all cases at day five and ranged from 40–60% by four weeks, with no difference between the control and treated groups. Of the adult worms recovered, the proportion of live worms did not differ significantly with exposure (Fig 1A and 1B). After eight weeks, recovery rates were slightly lower in groups exposed to FLBZ for shorter durations (6 and 12 hr); however, this was not significantly different from the 24 hr exposure (Fig 1B) or from control groups unexposed to the drug.

#### Motility

Motility was unaffected by FLBZ exposure. All recovered worms were highly motile five days following transplantation (S1 Fig). Motility was variable in worms recovered four and eight weeks post-transplantation, but motility impairment and drug exposure were not correlated (S1 Fig). A small proportion of worms were presumed dead due to a lack of motility.

#### Microfilarial release

**Fig 2:**
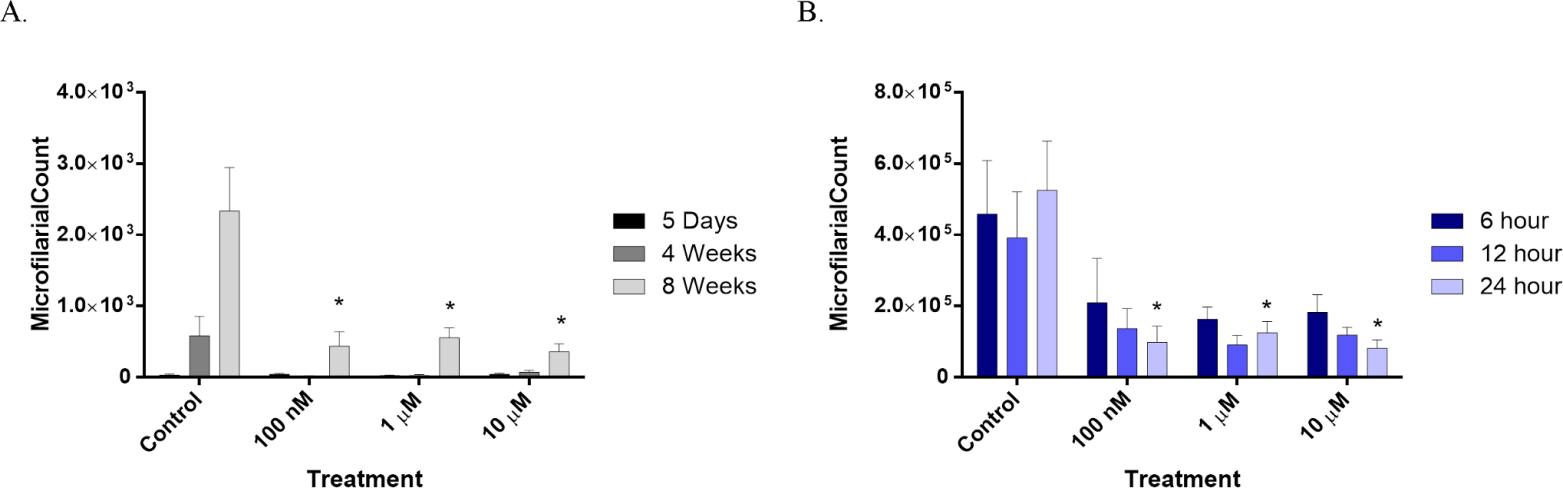
Effects of FLBZ on recovery of microfilariae from the peritoneal cavity of jirds. A. Five day, four week, or eight week maintenance after transplantation into naïve jirds following 24 hr culture. Data is a combination of two experiments. Microfilariae were enumerated from the peritoneal wash of each of three jirds which had 10 female worm transplanted in experiment one (5 day and 4 week maintenance) and 15 females in experiment two (4 and 8 week maintenance). Data were corrected for number of females transplanted. The 4 week data from each experiment did not differ significantly, therefore, they were combined for presentation purposes. B. Recovery eight weeks post-transplantation following 6, 12, or 24 hr exposure to FLBZ *in vitro*. Microfilariae were enumerated from peritoneal washes of each of three jirds which had 15 females worms transplanted, following the appropriate duration of *in vitro* exposure to FLBZ. Bars indicate the mean mf counts from each jird in a treatment group. Lines represent the standard deviation.

Five days following transplantation into naïve jirds, the number of mf recovered was consistently low and did not differ between control and treated groups (Fig 2A). At four weeks, there was an increase in mf abundance in the control groups, while the number remained low in FLBZ-exposed groups. At eight weeks, there was a significant increase in mf abundance in the control group and slight increases in the treated groups. Incubation time had no effect on mf abundance (Fig 2B). Significantly fewer mf were released from 24 hour treated females following 8 week *in vivo* maintenance (Fig 2B). This did not extend to the 6 and 12 hour exposures as there was a great degree of variability in mf counts from these groups.

### Embryograms

**Fig 3:**
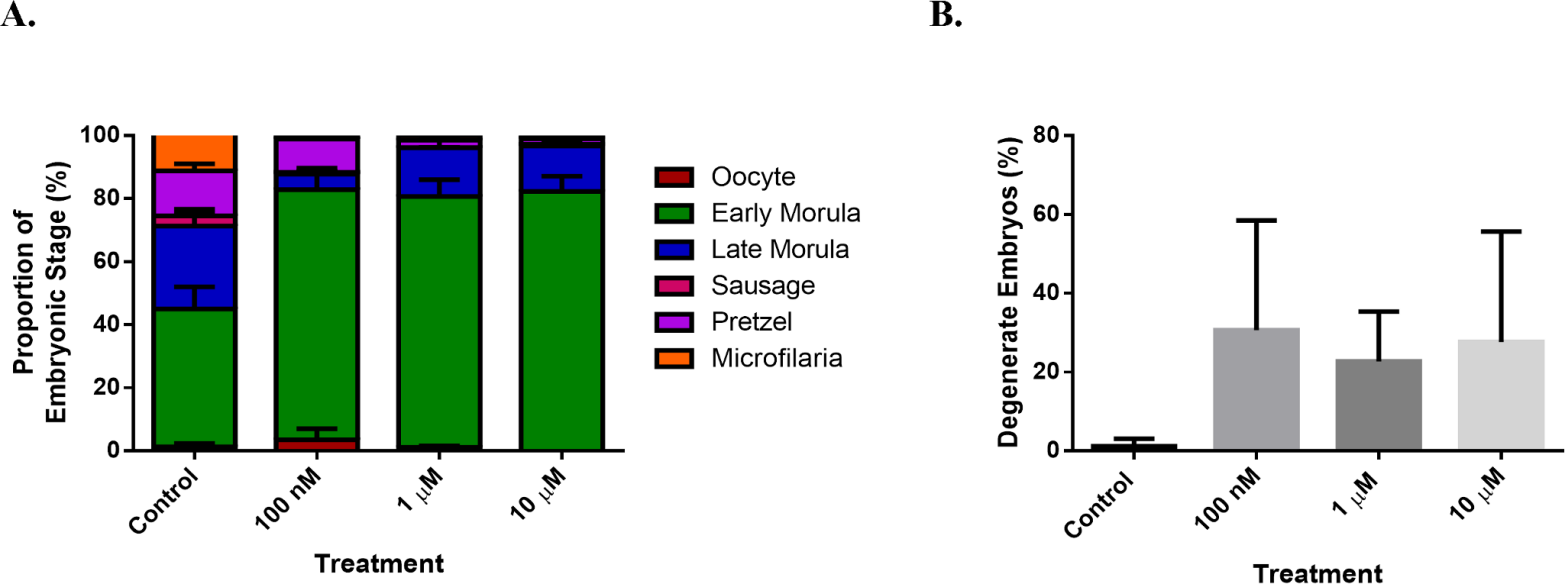
Effects of FLBZ on *B*. *malayi* embryogenesis. A. Embryograms for untreated and FLBZ-treated (24 hr exposure) females recovered four weeks post-transplantation. Two to four worms per treatment group were used for embryogram analysis. Data shown are % of oocytes, early and late morulae, pretzels and stretched mf in worm homogenates. Significantly more early developing embryos were observed in treated groups (*P* < 0.001). B. Proportion of degenerating intrauterine stages.

Embryograms of treated worms differed substantially from those of controls (Fig 3A). Late developing stages (sausage, pretzel and stretched mf) were markedly reduced, while early developing morula were significantly increased in treated worms relative to the controls. Treated worms not only contained large numbers of degenerating embryos (Fig 3B) they were also found to contain fewer embryos overall (S2 Fig).

### Effects of FLBZ on adult worm viability: Histology

**Fig 4:**
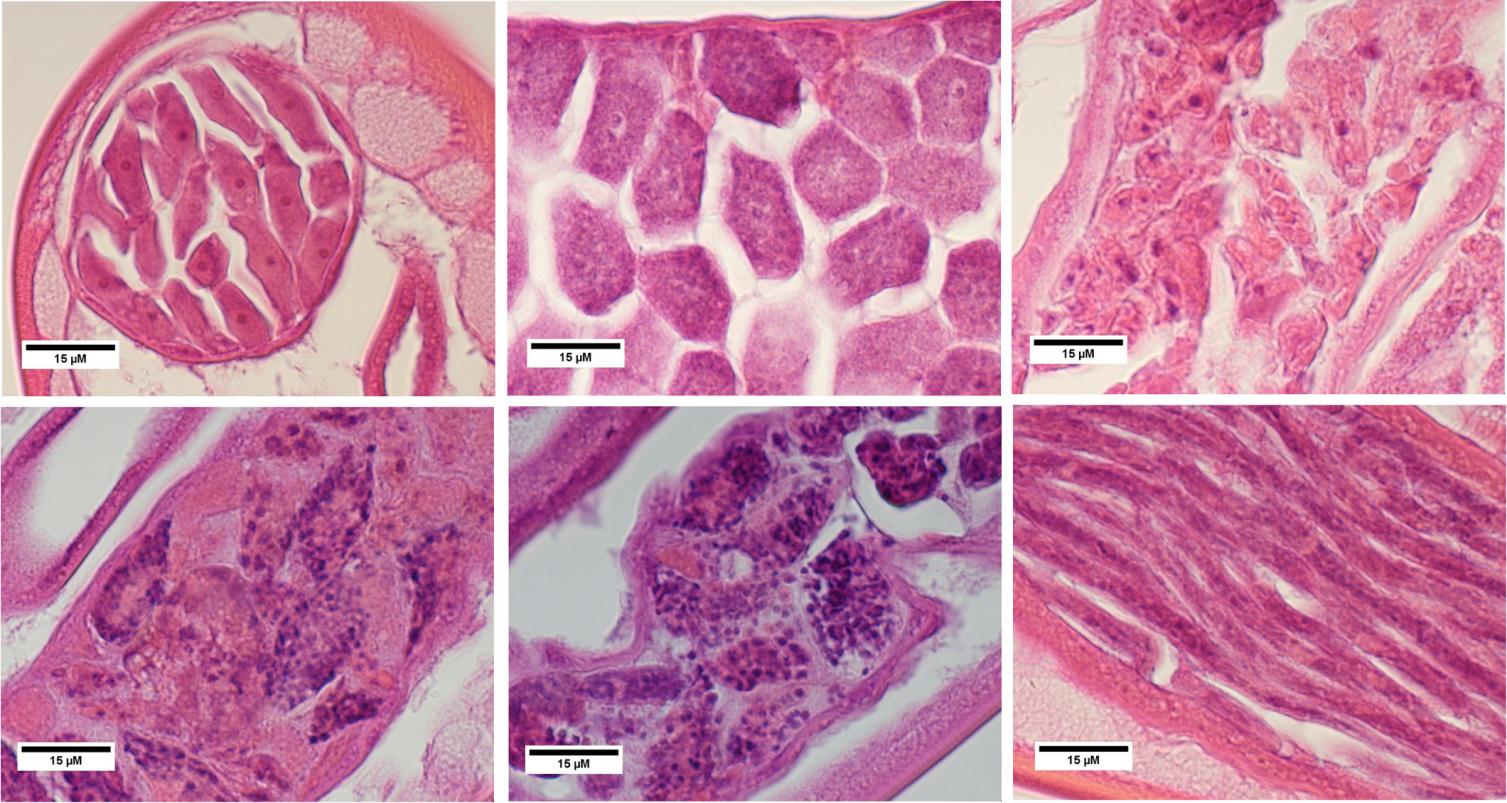
Long-term effects of FLBZ on developing embryos. Exposure period was 24 hr in culture prior to re-implantation. Time to recovery after transplant was 4 weeks. A. Oocytes, DMSO control; B. Morulae, DMSO control; C. Oocytes, 100 nM; D. Early Morulae, 1 μM; E. Late Morulae, 100 nM; F. Microfilariae, 10 μM.

Tissue damage observed in worms transplanted into naïve jirds differed from that observed in vitro [22]. Damage to the intestine observed *in vitro* was not observed following transplantation (Table 1). Minor damage to the hypodermis was observed five days following transplantation in both the treated and control groups; however, this damage appeared to resolve by four weeks. While the damage score in treated groups at four weeks returned a statistically significant p value, the rather low score indicates this effect may not be biologically relevant. Damage to the developing embryos was the most prominent effect. While extensive damage to embryos was observed in all treatments at five days, this damage appeared to resolve in the control group by four weeks. Damage to embryos of treated worms, however, was pronounced and did not resolve at four weeks. At this time point, damage to treated embryos was significantly higher than in the controls. Morula stage embryos were the most extensively damaged (Fig 4D and 4E) while mf were unaffected (Fig 4F).

**Table 1:** Tissue damage score following recovery of FLBZ-exposed *B*. *malayi* females from naïve jirds. A, B. Worms incubated for 24 hr prior to transplantation and recovered from jirds for 5 days, 4 weeks or 8 weeks after. Data is a combination of two experiments. In experiment one worms transplanted into each of three jirds for all treatment groups were maintained for 5 days or 4 weeks. Worms transplanted into each of three jirds per treatment group in experiment two were maintained for 4 weeks or 8 weeks. The 4 week data were combined for presentation purposes. A minimum of 25 worms were assessed histologically C, D. Worms incubated for 6, 12, or 24 hr prior to recovery at 8 weeks. Worms were transplanted into each of three jirds per treatment group, following the appropriate duration of *in vitro* exposure to FLBZ. The number of worms assessed histologically ranged from 11 to 39. Damage scored as minor (1), moderate (2), severe (3), or no damage (0) by two methods: A, C. Individual section scoring method; B, D. Classical histopathological survey method.

A		Hypodermis			Intestine			Early Embryo			Late Embryo		
		5 Day	4 Week	8 Week	5 Day	4 Week	8 Week	5 Day	4 Week	8 Week	5 Day	4 Week	8 Week
	Control	1.2	0.3	0	0.7	0.2	0.1	2	0.9	0	1.4	0.3	0.1
	100 nM	1.1	*0.6	0.1	0.3	0.3	0.2	2	*1.7	0.6	1.2	*0.9	0.6
	1 uM	1.5	*0.6	0.1	0.4	*0.4	0	2.3	*1.9	0.9	1.6	*1.2	0.6
	10 uM	1.3	*0.6	0	0.3	0.2	0	1.8	*1.9	0.5	2	*0.9	0.2
B		Hypodermis			Intestine			Early Embryo			Late Embryo		
		5 Day	4 Week	8 Week	5 Day	4 Week	8 Week	5 Day	4 Week	8 Week	5 Day	4 Week	8 Week
	Control	0.4	0	0.7	0	0	0	1.9	0.8	1	1.2	0.6	0
	100 nM	0.7	0.2	0.3	0.2	0.2	0	1.7	1.7	1	1.4	1.9	0
	1 uM	0.5	0	0	0	0	0	1.4	1.4	1	1.3	1.8	0
	10 uM	0.4	0.2	0	0	0	0	1	1.8	1.6	2	1.8	0
C		Hypodermis			Intestine			Early Embryo			Late Embryo		
		6 hr	12 hr	24 hr	6 hr	12 hr	24 hr	6 hr	12 hr	24 hr	6 hr	12 hr	24 hr
	Control	0	0	0	0.1	0	0.1	0.9	1.3	0	0.4	0.2	0.1
	100 nM	0.1	0.1	0.1	0.2	0.1	0.2	0.6	1.2	0.6	0.1	0.2	0.6
	1 uM	0.1	0	0.1	0.2	0.1	0	0.4	0.5	0.9	0.4	0	0.6
	10 uM	0	0.1	0	0.1	0.1	0	0.4	0.7	0.5	0.3	0.1	0.2
D		Hypodermis			Intestine			Early Embryo			Late Embryo		
		6 hr	12 hr	24 hr	6 hr	12 hr	24 hr	6 hr	12 hr	24 hr	6 hr	12 hr	24 hr
	Control	0	0	0.7	0.7	0	0	0.9	1.2	1	0	0	0
	100 nM	0	0	0.3	0.3	0.3	0	1.2	1	1	0	0	0
	1 uM	0	0.5	0	1	0	0	1.7	0.5	1	0	0	0
	10 uM	0	0	0	0.7	0	0	1	1.5	1.6	0	0	0

*p-value <0.05

## Discussion

An important step in the development of an anthelmintic is identifying the exposure profile (concentration and duration of exposure) which leads to death or irreversible damage in culture. These data can be used to predict efficacious pharmacokinetic (PK) patterns, leading to more efficient selection of doses for clinical trials in diseases, such as the human filariases, that have long end points, based on PK data rather than efficacy. This is particularly true for FLBZ, which has little apparent acute toxicity for adult filariids in culture [18,22], but is highly efficacious when administered to infected animals in parenteral regimens that afford prolonged exposure to low blood levels [18]. Replicating this exposure pattern necessitates long-term parasite maintenance in culture, which has been difficult to achieve without the presence of feeder cells that may compromise the integrity of added drugs.

Efforts to reformulate FLBZ to provide an orally bioavailable macrofilaricide necessitate replication of the efficacy achieved in a “long, low” exposure profile in a “high, short” paradigm. To determine if short exposures (~1 day) to high but pharmacologically relevant concentrations of FLBZ can cause lethal damage to adult filariids, we implemented an *in vivo* protocol in which the long-term effects of short-term exposure to FLBZ on *B*. *malayi* can be determined by transplanting treated worms into naïve jirds following drug exposure. The choice of FLBZ concentration and duration of exposure in this study was based on pharmacokinetic studies in rats, mice and pigs[20,27–29]. In rats, a cyclodextran oral formulation resulted in a maximum plasma level of approximately 7 μM and remained at a concentration of >1 μM for 6 hours [20]. Dosing pigs with a cyclodextran oral formulation saw FLBZ in the plasma for longer durations, similar to that of the 12 hour time point in this study, albeit at lower concentrations [27]. The concentrations chosen have also been shown to elicit detrimental effects on adult females over short-term *in vitro* incubations [22].

Several important conclusions can be drawn from this study. First, adult filariids have the capacity to recover from damage. This is demonstrated by the observation that control worms recovered 5 days after transplantation showed clear signs of tissue damage; however, damage was not evident in control worms recovered 4 or 8 weeks post-transplantation, suggesting that the process of removal from the initial jird host and maintenance in culture for 24 hr is traumatic, but that the organisms can recover.

Second, exposure to FLBZ for 6–24 hr is deleterious to adult filariids; female *B*. *malayi* exposed to the drug in culture and recovered 4 weeks after transplant exhibited considerable tissue damage, especially to reproductive tissues. These worms were unable to produce mf.

Third, adult *B*. *malayi* are resilient to drug-induced damage; worms recovered 8 weeks after transplantation had generally resumed production of mf and had resolved the damage observed at 4 weeks after transplantation. This degree of recovery was unanticipated and suggests the possibility of a more sophisticated and robust healing response to injury in these organisms than we had anticipated.

Worm recovery from control groups was comparable to recovery rates obtained in earlier studies [30,31]. FLBZ exposure had no effect on recovery of adult worms. This result suggests that 24 hr incubation in up to 10 uM FLBZ does not cause irreversible damage that leads to worm death within eight weeks following exposure.

How FLBZ eliminates adult filariae following parenteral dosing *in vivo* remains unknown. Damage from prolonged, low-concentration exposure to FLBZ following parenteral administration results in slow killing in weeks to months [16–19]; efficacy may require an immune response as is thought to be the case for microfilaricidal agents [23,32,33]. It remains a goal of reformulation efforts to recapitulate the high efficacy of parenteral FLBZ with an oral regimen. The current results suggest that adult female *B*. *malayi* can recover from a short exposure to FLBZ, but leave unanswered the question of whether oral regimens compatible with field use of a macrofilaricide (up to 7 consecutive days) can cause lethality.

It is evident that the process of transplantation is stressful to the worms, as we observed damage to control worms recovered 5 days post-transplantation; however, they are able to recover from this injury (Table 1). While control worms recovered from the transplantation process, FLBZ-exposed worms were less able to do so (Table 1), especially in reproductive tissues, suggesting that they are indeed compromised by the drug.

FLBZ damages the hypodermis, intestine and developing embryos in adult female *B*. *malayi* exposed to the drug in culture [22]. In the present study, drug damage to the intestine or hypodermis was resolved after longer residence times in the host. In an early study which injected FLBZ parenterally, no alterations in intestinal cells were reported in recovered worms beyond a decrease in microtubules until the experiment ended at day six [34]. It was suggested this was due to the limited role of the intestine in nutrient acquisition by filariid parasites; unfortunately, damage to the hypodermis, which also plays a role in nutrient acquisition, was not reported. That this earlier work confirmed the lack of damage observed in the intestine and hypodermis in the present study does not mean that effects on these tissues can play no role in the macrofilaricidal activity of FLBZ. While hypodermal damage was not observed, this previous study reported a loss of intestinal microtubules, which likely plays a role in the extensive damage to the hypodermis observed *in vitro* [22]. However, it cannot be ignored that exclusion of the host at the time of anthelmintic exposure is a limitation to *in vitro* culture systems as it overlooks an important component: the host response. Since we exposed parasites to FLBZ prior to transplantation into a naïve jird, an important factor may be missing in the development of drug-induced tissue damage.

Consistent among FLBZ studies is the damage caused to developing embryos [22,34,35]. In this study, the embryonic stage which displayed the greatest damage was the morula (Table 1, Fig 4). Disruption to the integrity of morulae is prominent in treated worms which exhibited an apparent loss of cellular adhesion and dispersion of these cells (Fig 4D and 4E). Similar results were observed in *O*. *gibsoni* infected cattle administered five daily doses of mebendazole (MBZ); two weeks following treatment, degenerating morula was the most notable effect [36]. They also report the presence of various developmental stages in the same uterine section, consistent with our results where early morula are found alongside degenerating morula (Fig 4D). Four weeks following treatment degenerate morula were mixed with normal mf. Mf exhibited normal appearance for as long as eight weeks post exposure and were found mixed with oocytes and embryonic debris [36]. This degeneration of morula correlated with an increase in released egg antigen that is not observed with exposure to microfilaricidal drugs IVM and DEC [36].

Embryograms indicate that morulae were also the most abundant stage, which increased with treatment and coincided with a decrease in the proportion of later developmental stages (sausage, pretzel, mf; Fig 3). Exposure of filariae to anti-*Wolbachia* antibiotics resulted in a similar phenomenon, whereby the proportion of later developmental stages decreased with increasing drug exposure [37]. No previous studies have documented changes in filarial embryogram profiles associated with benzimidazole exposure. However, histological analysis of nodules from human onchocerciasis patients treated intramuscularly with FLBZ revealed that females contained only oocytes and small numbers of mf two month following treatment. The Forsyth [36] and Dominguez-Vazquez [19] studies both administered drug over a period of five days. While we see similar trends in exposed embryos, this suggests that multiple doses, rather than single exposures as in this study, may be required for high efficacy.

That there was no damage to stretched intrauterine mf at any time point is not surprising, as this observation is consistent with other studies [16,22,30,38]. In view of the fact that mf are in an arrested developmental state, there is likely to be a reduced requirement for microtubule-dependant processes with which FLBZ may interfere. However, there was a concurrent impairment of mf release, measured as reduced abundance of mf recovered from the peritoneal cavity (Fig 2). At eight weeks, there was a drastic increase in mf abundance in the control group compared to the treatment groups. There was a slight rebound in mf numbers in treated groups between 4 and 8 weeks after transplantation. Diminution in released mf could result from uterine blockage due to degenerating embryos or an inability of females to release mf. Alternatively, if *B*. *malayi* embryos follow the same developmental cycle as *Onchocerca volvulus* [26], it may be that embryos present at treatment were damaged and newly developed oocytes were unable to mature to mf. The potential for embryos to recover, or for the females to resume normal embryo production, is yet to be determined. Nevertheless, the limited effect observed on mf is encouraging as it supports the potential safety of FLBZ for use in *L*. *loa* endemic regions. MDA campaigns for onchocerciasis in Africa are limited due to the activity of ivermectin against *L*. *loa* mf and association with severe adverse events [11]. An ideal drug for onchocerciasis would have little effect on mf in macrofilaricidal regimens.

The present findings demonstrate that FLBZ elicits detrimental effects on developing embryos following long-term maintenance in the peritoneal cavity of jirds. Damage was most evident in early developmental stages and resulted in a decreased output of stretched mf, which is presumed to limit transmission. Conversely, FLBZ did not have direct microfilaricidal effects, which had implications for the utility of FLBZ in areas co-endemic for *L*. *loa*. This is an important observation, as current MDA programmes are restricted by the SAEs resulting from rapid mf killing by existing drugs. If FLBZ is shown to have a similar lack of microfilaricidal effect on *L*. *loa*, it could further substantiate its utility as a macrofilaricide in *Loa* endemic regions. It will, therefore, be critical to determine the effects of FLBZ on *L*. *loa* mf.

## Supporting Information

S1 Fig Motility of adult worms recovered from the peritoneal cavity of *Brugia malayi* infected jirds. A. Motility of adults worms from the first experiment where worms were maintained *in vivo* for five days or four weeks following 24 hour *in vitro* exposure to FLBZ. B. Motility of adults worms from the second experiment where worms were maintained *in vivo* for four weeks or eight weeks following 24 hour *in vitro* exposure to FLBZ. We also report motility for eight week *in vivo* maintained worms following 12 and 6 hour *in vitro* exposure.
(TIF)

S2 Fig Average total number of embryonic developmental stages in in adult females recovered from the peritoneal cavity of *Brugia malayi* infected jirds four weeks post-transplantation. Numbers are averages of counts from homogenates of a minimum of six female worms per treatment.
(TIF)
