# Supplementary figures and images for: An In Vitro/In Vivo Model to Analyze the Effects of Flubendazole Exposure on Adult Female Brugia malayi
Source: PLoS Negl Trop Dis. 2016 May 4;10(5):e0004698. doi: 10.1371/journal.pntd.0004698 (PMC4856366; doi:10.1371/journal.pntd.0004698)

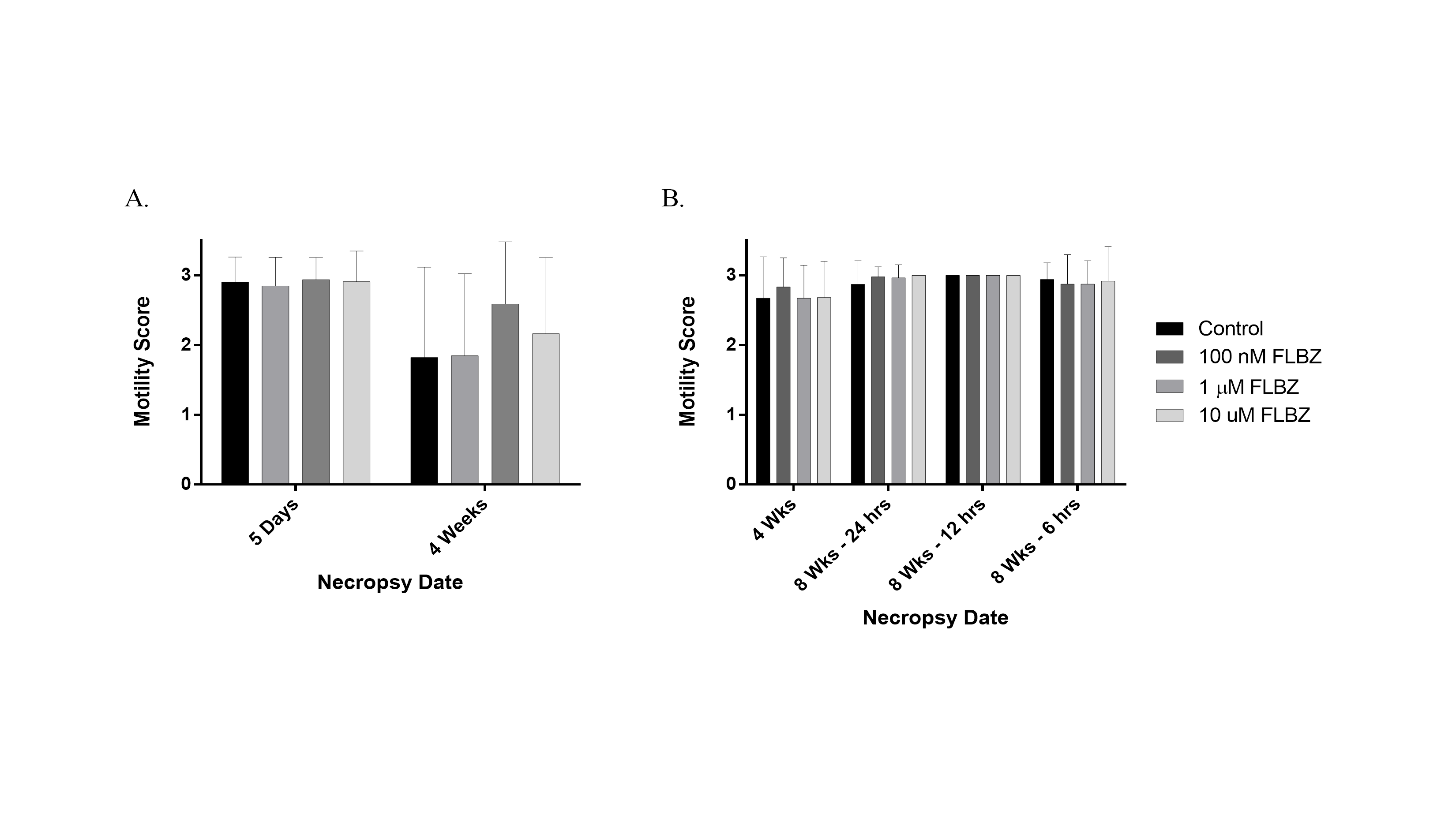

Supplement: S1 Fig — A. Motility of adults worms from the first experiment where worms were maintained in vivo for five days or four weeks following 24 hour in vitro exposure to FLBZ. B. Motility of adults worms from the second experiment where worms were maintained in vivo for four weeks or eight weeks following 24 hour in vitro exposure to FLBZ. We also report motility for eight week in vivo maintained worms following 12 and 6 hour in vitro exposure. (TIF) [file pntd.0004698.s001.tif]

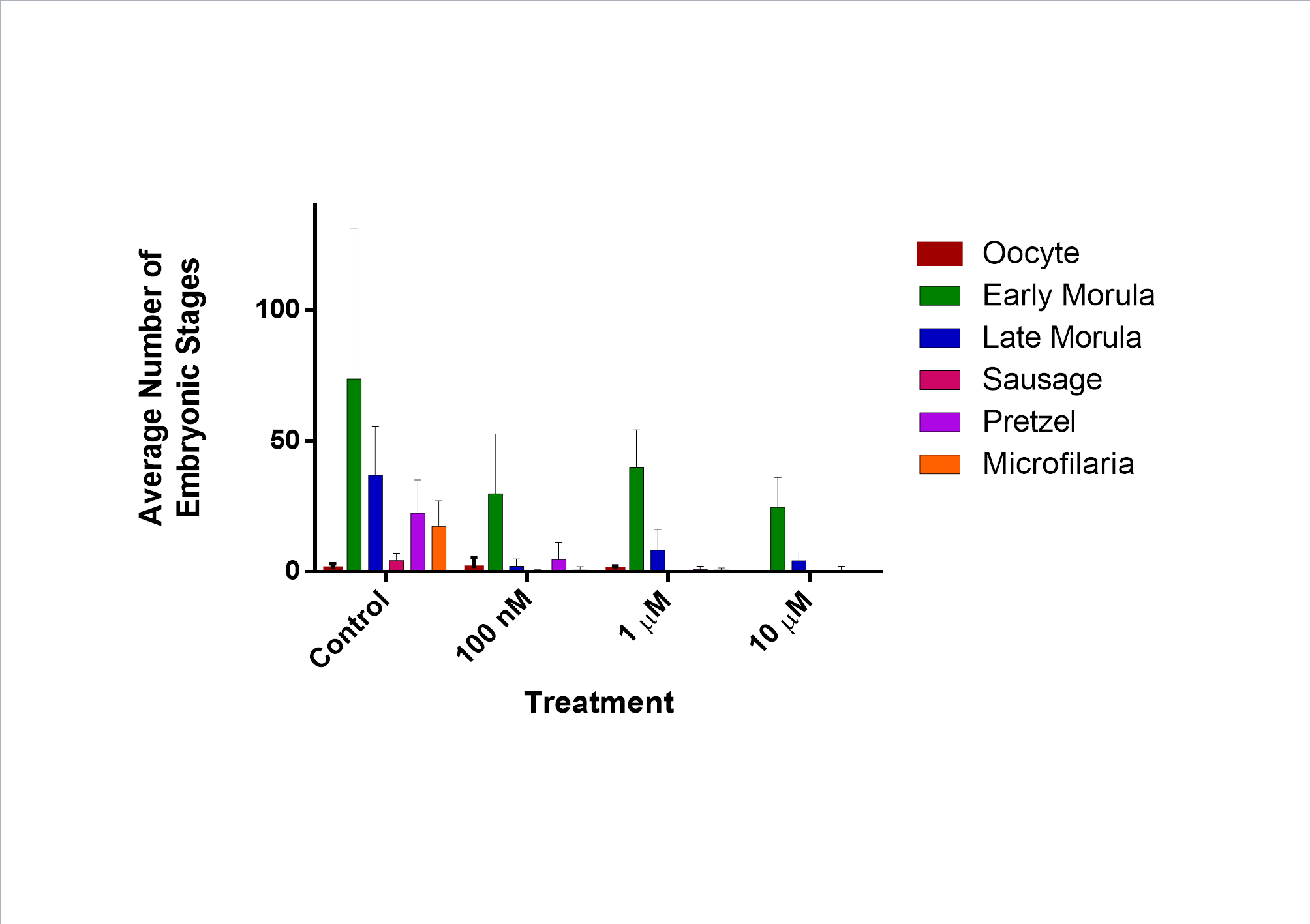

Supplement: S2 Fig — Numbers are averages of counts from homogenates of a minimum of six female worms per treatment. (TIF) [file pntd.0004698.s002.tif]
